# Acute Confusional Syndrome and Covid-19 disease. Clinical and Sociodemographic differences with other comorbid diseases

**DOI:** 10.1192/j.eurpsy.2022.1227

**Published:** 2022-09-01

**Authors:** D. García Hernández, M. Calls Samora, A. Llimona González, F. Dinamarca, F. Casanovas, A. Pérez Oms, C. Llimona Sánchez, S. Oller Canet

**Affiliations:** 1 Parc de Salut Mar, Instituto De Neuropsiquiatría Y Adicciones (inad), Barcelona, Spain; 2 Parc de Salut Mar, Institut De Neuropsiquiatria I Addiccions (inad), Barcelona, Spain

**Keywords:** delirium, ACUTE CONFUSIONAL SYNDROME, Covid-19

## Abstract

**Introduction:**

Coronavirus Disease 19 (COVID-19) was declared a pandemic by the World Health Organization (WHO) in March 2020. Since the outbreak, neuropsychiatric presentations such as delirium have been developing.

**Objectives:**

Our aim is to describe sociodemographic and clinical differences between inpatients cursing with Acute Confusional Syndrome (ACS) with and without COVID-19 pneumonia.

**Methods:**

This is an observational-descriptive study. All patients attended by the liaison psychiatry service of Hospital del Mar, between February and April 2020, with ACS diagnosis were included. The sample was divided in 2 groups (with and without COVID-19 pneumonia). Sociodemographic and clinical variables including sex, age, previous somatic or psychiatric history, ACS risk factors, ACS subtype and pharmacological treatment were compared. Chi-square and U Mann Whitney tests were used.

**Results:**

The total sample was 62 patients. 43.5% were women with a mean age of 71,7 (SD 11,3). Covid pneumonia group included 26 patients. There was a higher percentage of Hypoxemia in Covid pneumonia patients (p<0,001). There were significant differences between Covid pneumonia group and ACS in relation to: a previous diagnosis of Ischemic Heart Disease (p=0,007), Heart Failure (p=0,029) and Nephropathy (p=0,022). Dexmedetomidine (p=0,001) was highly used for ACS treatment in Covid pneumonia patients.

**Conclusions:**

In this sample, patients with ACS and Covid pneumonia had a bigger rate of hypoxemia and previous history of Ischemic Heart Disease, Heart Failure and Nephropathy compared to the rest of ACS patients. Dexmedetomidine was more commonly used for the treatment of ACS in Covid pneumonia group. More studies would be necessary to assess the significance.

**Disclosure:**

No significant relationships.

